# Obtunding Sensitive Dentine

**Published:** 1880-01

**Authors:** 


OBTUNDING SENSITIVE DENTINE.—Prof. C. N. Pierce, of the Pennsylvania College of Dental Surgery, gives, in the November No., '79, *Dental Cosmos*, the following new obtunder, and he suggests, as worthy of trial: "Glacial phosphoric acid, dissolved in two parts of water by weight and subsequently evaporated *rapidly* by heat to the consistency of glycerine. The reason for directing its *rapid* evaporation is that if the operation is conducted slowly the solution will crystallize on cooling—a result which does not occur when treated as suggested. The sensitive spot, after thorough drying, should be touched with this solution. It has proved more efficient in a larger number of cases than any other material which I have tried. It is especially satisfactory when applied to the necks of the teeth, where there is exposure of the cementum and dentine, and the exalted sensitiveness which frequently accompanies such exposure. In cases of severe pain from the wounding of an exposed pulp, or following the application of the arsenical paste, let any foreign substance be removed, the cavity dried, and then, upon a small pledget of cotton on the point of an instrument, carry a drop of the liquid to the injured part. Relief will in many cases be almost instantaneous. Such has been the experience of the writer."

